# Qualitative Evidence Synthesis (QES) for Guidelines: Paper 2 – Using qualitative evidence synthesis findings to inform evidence-to-decision frameworks and recommendations

**DOI:** 10.1186/s12961-019-0468-4

**Published:** 2019-08-08

**Authors:** Simon Lewin, Claire Glenton, Theresa A. Lawrie, Soo Downe, Kenneth W. Finlayson, Sarah Rosenbaum, María Barreix, Özge Tunçalp

**Affiliations:** 1 Norwegian Institute of Public Health, Oslo, Norway; 2 Health Systems Research Unit, South African Medical Research Council, Cape Town, South Africa; 3 Evidence-based Medicine Consultancy, Bath, United Kingdom; 4 University of Central Lancashire, Preston, United Kingdom; 5 Department of Reproductive Health and Research including UNDP/UNFPA/UNICEF/WHO/World Bank Special Programme of Research, Development and Research Training in Human Reproduction (HRP), World Health Organization, Geneva, Switzerland

**Keywords:** evidence-to-decision, guideline development, GRADE, GRADE-CERQual, QES, qualitative review, qualitative evidence synthesis, qualitative methods, WHO guidelines

## Abstract

**Background:**

WHO has recognised the need to improve its guideline methodology to ensure that guideline decision-making processes are transparent and evidence based, and that the resulting recommendations are relevant and applicable. To help achieve this, WHO guidelines now typically enhance intervention effectiveness data with evidence on a wider range of decision-making criteria, including how stakeholders value different outcomes, equity, gender and human rights impacts, and the acceptability and feasibility of interventions. Qualitative evidence syntheses (QES) are increasingly used to provide evidence on this wider range of issues. In this paper, we describe and discuss how to use the findings from QES to populate decision-making criteria in evidence-to-decision (EtD) frameworks. This is the second in a series of three papers that examines the use of QES in developing clinical and health system guidelines.

**Methods:**

WHO convened a writing group drawn from the technical teams involved in its recent (2010–2018) guidelines employing QES. Using a pragmatic and iterative approach that included feedback from WHO staff and other stakeholders, the group reflected on, discussed and identified key methods and research implications from designing QES and using the resulting findings in guideline development.

**Results:**

We describe a step-wise approach to populating EtD frameworks with QES findings. This involves allocating findings to the different EtD criteria (how stakeholders value different outcomes, equity, acceptability and feasibility, etc.), weaving the findings into a short narrative relevant to each criterion, and inserting this summary narrative into the corresponding ‘research evidence’ sections of the EtD. We also identify areas for further methodological research, including how best to summarise and present qualitative data to groups developing guidelines, how these groups draw on different types of evidence in their decisions, and the extent to which our experiences are relevant to decision-making processes in fields other than health.

**Conclusions:**

This paper shows the value of incorporating QES within a guideline development process, and the roles that qualitative evidence can play in integrating the views and experiences of relevant stakeholders, including groups who may not be otherwise represented in the decision-making process.

**Electronic supplementary material:**

The online version of this article (10.1186/s12961-019-0468-4) contains supplementary material, which is available to authorized users.

## Background

Decision-makers typically have a range of questions when deciding whether to recommend or implement a particular health intervention, including the effectiveness of the intervention, its acceptability and feasibility, equity impacts and the resources needed for implementation [1]. Efforts to address these questions have led to interest across a number of settings, including within guideline development agencies, in expanding the evidence base used to inform decisions on health interventions [2]. Using a broader range of evidence may help to ensure that decisions are relevant and applicable.

As a guideline producing organisation, WHO has recognised the need to improve its guideline methodology to ensure that these processes are transparent and evidence based, and that the resulting recommendations are relevant and applicable [3, 4]. To help support this, the WHO Handbook for Guideline Development now stipulates that evidence on a number of questions is required to inform a WHO guideline recommendation [4]. These questions include how people affected by the intervention value different outcomes, the effectiveness, acceptability and feasibility of the intervention, and equity implications. Along with other organisations, WHO increasingly uses the GRADE evidence-to-decision (EtD) framework for this purpose [5, 6]. The EtD framework helps to ensure that key questions or criteria are considered in decisions, and also supports people in assessing and using evidence in a more systematic, structured and transparent way. Evidence is compiled from systematic reviews and other sources to address each of the framework’s criteria [5] (Additional file 1).

As discussed in paper 1 in this series, to address EtD framework criteria such as the acceptability and feasibility of interventions, guideline producers are now exploring the use of qualitative evidence [7, 8]. This has led to growing interest in systematic reviews of qualitative studies (also known as qualitative evidence syntheses (QES)) – an approach for synthesising the findings from multiple primary qualitative studies. Like systematic reviews of the effectiveness of interventions, QES can provide key evidence for informing guideline recommendations and other decisions [2, 7, 8].

The first WHO guideline to draw systematically on findings from QES was produced by the WHO Department of Reproductive Health and Research in 2012 [9]. Since then, a number of guidelines have been published using this approach [10–15], and others are in preparation. In these guidelines, QES findings have provided evidence on how people value different outcomes, on the acceptability and feasibility of interventions, and on equity impacts. Additionally, in at least two guidelines [11, 13], a priori QES were undertaken at the guideline scoping stage to determine what outcomes were important to the group that was the primary focus of the guideline.

**Fig. 1:**
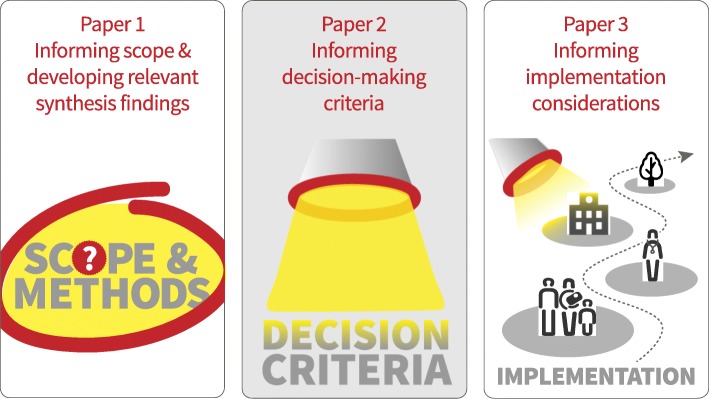
Overview of the ‘Qualitative evidence synthesis in guidelines’ series of papers

This paper is the second of a series of three papers that describe and discuss the use of QES to inform the development of clinical and health system guidelines (Fig. 1). The first paper deals with how QES findings can inform the scope of a guideline and be used to develop Summary of Qualitative Findings statements for key guideline decision-making criteria [16]. The third paper deals with how QES findings can inform implementation considerations included in guidelines [17]. Throughout the series, we explore the strengths and limitations of these approaches, provide examples of what worked and what was less successful, and make suggestions for improvements.

## Aim of this paper

The aim of this paper is to describe and discuss how findings from QES can be used to populate key EtD framework criteria for decision-making in guideline development and to inform recommendations. As members of technical teams responsible for producing evidence for WHO guidelines, we describe lessons learnt from our experiences and areas in which further research and development are needed.

## Methods

The experiences, guidance and data presented in this series of papers are the result of a range of processes that have evolved over a decade of engagement with qualitative research in the context of developing healthcare guidelines at WHO. To develop this series of papers, we used a pragmatic and iterative approach that included the following steps:

- WHO convened a core team of authors who had been involved in WHO guideline technical teams since 2010 and in developing QES to support these guidelines. The team included people with extensive experience in qualitative research and qualitative evidence synthesis methods, methods for guideline development and the use of evidence-to-decision frameworks.
- The core author team reflected on the guideline development processes in which we had been involved (see list below), focusing on the role of QES findings in these processes. We also received informal feedback on these processes from other WHO staff involved in guideline development, and from participants in several guideline training workshops at WHO. These reflections and feedback led us to identify three key areas that each became a focus for one of the papers in the series, namely how QES methods need to be adapted for the context of producing guidelines; how to use findings from QES to populate EtD frameworks; and how to use QES findings to develop implementation considerations and inform implementation guidance and processes.
- The lead author for each paper then drafted an outline for their paper, and these were discussed during a 4-day author workshop. In the workshop, authors discussed the most important factors in the use of qualitative evidence in this context to date and agreed on what worked and what could be improved in the future. The outlines were then developed into full papers, using an iterative process of sequential writing and discussion. We also identified relevant examples from the guidelines in which we had been involved. The core authors then reviewed the draft to clarify the ideas and processes described and to add further examples where needed.
- We then circulated the draft papers to key stakeholders to obtain their feedback on the ideas and processes described. These stakeholders included members of WHO guideline panels (sometimes called Guideline Development Groups), methodologists, guideline commissioners and implementation experts.

We selected examples from the following WHO guidelines in which members of the core author team had been involved:

1. Optimizing health worker roles for maternal and newborn health through task shifting (2012) [9]
2. Expanding health worker roles to help improve access to safe abortion and post-abortion care (2015) [10]
3. WHO recommendations on antenatal care for a positive pregnancy experience (2016) [11]
4. WHO recommendations on intrapartum care for a positive childbirth experience (2018) [13]
5. Guidance on communication interventions to inform and educate caregivers on routine childhood vaccination in the African Region (World Health Organization Regional Office for Africa: Guidance on Communication Interventions to Inform and Educate Caregivers on Routine Childhood Vaccination in the African Region, forthcoming)
6. WHO recommendations on digital interventions for health systems strengthening [18]

All of these guidelines were health systems focused or had a health system component, and all used the GRADE EtD frameworks [6]. As alluded to above, the frameworks are documents with a common structure that includes a question, an assessment of the evidence that addresses the question, and a conclusion, which facilitate explicit and transparent decision-making [5]. We selected examples in this paper to highlight the use of qualitative evidence in the guideline processes described, including the strategies used to package this evidence for decision-making. In some cases, we have made small changes to the examples so that they can stand alone from the guideline text or to ensure that they better show the issue they are intended to highlight. We have noted in the text where we have adapted examples from published guidelines.

## Results

### Using findings from qualitative evidence syntheses to populate EtD frameworks and other similar decision support tools

In a WHO guideline, the technical team creates EtD frameworks for each guideline question. The team then uses relevant evidence to populate each of the framework’s criteria (effectiveness, resource use, acceptability, feasibility and equity). These frameworks are the main documents used by the guideline panel during the final guideline meeting. Here, guideline panel members are asked to assess and make judgements about the evidence for each of these criteria before making a recommendation.

**Fig. 2:**
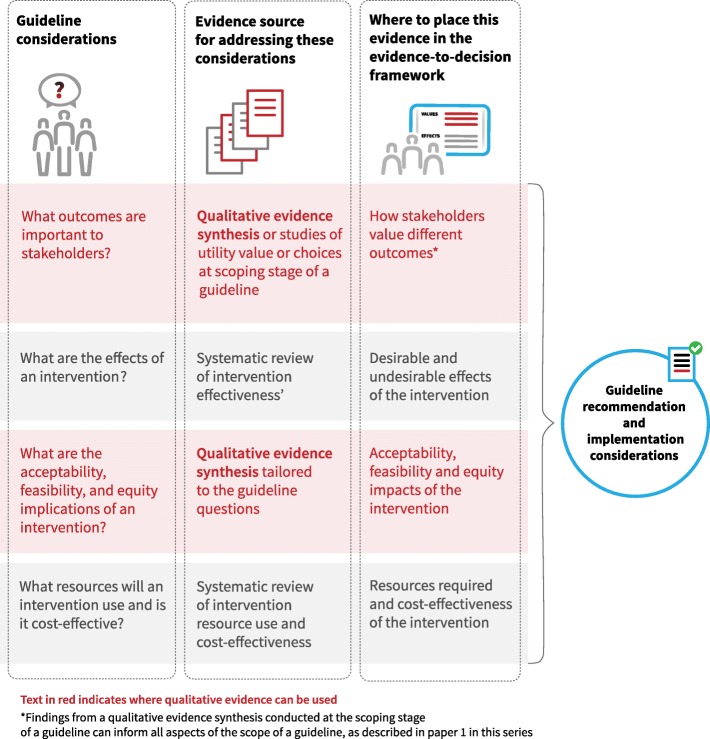
Where qualitative evidence can be used in relation to the GRADE evidence-to-decision framework criteria

Figure 2 and Table 1 show where qualitative evidence can be used in relation to the criteria in the EtD framework. We discuss below how to populate the framework for each of these criteria, apart from implementation considerations, which are discussed in paper 3 of this series [17].

**Table 1:** Criteria of the GRADE evidence-to-decision framework and where qualitative evidence might be useful in relation to these criteria

Criteria that are typically considered in GRADE evidence-to-decision frameworks	Where qualitative evidence may be useful and what type
How large are the positive (desirable) effects of the intervention?	Not applicable
How large are the negative (undesirable) effects of the intervention?	Not applicable
What is the overall certainty of the evidence of effects?	Not applicable
Is there important uncertainty about or variability in how much people value the outcomes and/or interventions?	QES at the scoping stage of the guideline or decision process^a^
What is the overall balance of effects?	QES findings on how the key stakeholder groups, including citizens, service users and service providers, value different outcomes
How large are the resource requirements?	Not applicable
What would be the impacts on gender, health equity and human rights?	QES findings on equity issues such as barriers and facilitators to accessing the option
Is the option acceptable to key stakeholders?	QES findings on the acceptability of the option
Is the option feasible to implement?	QES findings on the feasibility of the option
What are the implementation considerations?	QES findings that informed the other framework criteria can be used to develop or infer implementation considerations^b^

^a^Using QES findings at the scoping stage of a guideline is discussed in paper 1 in this series [16]

^b^How the findings from QES can be used to develop or infer implementation considerations is discussed in paper 3 in this series [17]

### Identifying relevant qualitative evidence

Findings from a QES may enter a guideline process in two ways:

- Through already-published syntheses that address the guideline questions directly or indirectly
- Through one or more syntheses commissioned for the guideline (Box 1). These may include both broad QES covering multiple guideline interventions and ‘mini-QES’ focusing on a specific intervention

Undertaking simple searches for relevant syntheses early in the guideline process may help the technical team decide whether it is necessary to commission new syntheses. For example, the technical team could search a database of systematic reviews in health, such as Epistemonikos (www.epistemonikos.org), for a limited time period, for instance, the last 5 years. A judgement on whether new syntheses need to be commissioned could then be made based on the syntheses identified (if any), including their scope, the synthesis approaches used and when the syntheses were conducted.

Syntheses used in a guideline may focus on people’s views regarding the interventions addressed by the guideline such as communication interventions in labour. Syntheses may also focus on the problem or issue underlying the interventions being addressed by the guideline, for instance, the ways in which women and healthcare providers communicate during labour. Syntheses may also include evidence that that is more, or less, direct or relevant, in relation to the guideline question. For example, a synthesis may focus on the views of people in a specific context, such as primary healthcare, while the guideline may include all levels of healthcare. Such differences are taken into account when assessing confidence in the evidence using the GRADE-CERQual approach. Guidance on applying the CERQual approach is available elsewhere [19, 20].

Syntheses vary in how their findings are presented, depending on whether a more aggregative or interpretive synthesis method is used [21], on whether thick or in-depth data underlie a synthesis finding, and on the review authors’ writing style. Where a synthesis aims to provide explanations or build theory, the findings may be presented both narratively and figuratively, for example, in the form of an infographic or logic model [22]. These infographics and logic models can be incorporated into an EtD where appropriate, for example, where they help to explain factors affecting the acceptability of an intervention. Although a large number of QES include infographics and logic models (e.g. [23, 24]), we have few examples of their inclusion in EtD [25].

In the discussion that follows, we assume that findings come from well conducted QES and that each finding is accompanied by an assessment of confidence using the CERQual approach. An assessment of confidence in or certainty of the evidence is required by a number of guideline development agencies, including WHO, to ensure that those making recommendations can take into account both the review finding and information on confidence in that finding [4]. CERQual is ideally applied at the time of conducting a synthesis but can also be applied post-hoc by the guideline technical team [26].

### Populating evidence-to-decision framework criteria with qualitative evidence – principles and processes

Once the draft findings from a QES are available, the next step is to package these findings for the relevant EtD framework criteria (Table 1). The nature of this type of evidence means that it does not always fit well within the summary-based and compartmentalised structure of the EtD framework. This may also be an issue where the technical team use findings from QES that were not undertaken specifically for the guideline. We discuss below some of the strategies that guideline technical teams can use to manage this.

When using QES findings to populate an EtD framework, technical teams may have queries regarding the meaning or scope of a finding or regarding the CERQual assessment. Ongoing interaction between the technical and QES teams is desirable to address these queries and may result in a finding being reformulated or the CERQual assessment being adjusted, or even a new search and mini-review being undertaken. For instance, in the WHO intrapartum care guideline an additional QES on pharmacological and nonpharmacological pain relief methods for childbirth was undertaken to supplement the wider intrapartum care QES [13].

#### Allocate the findings to the different criteria in the EtD frameworks

A QES finding may be relevant to more than one criterion (for instance, to both intervention acceptability and feasibility) and sometimes a pragmatic decision will need to be taken on where to place the finding. Overall, the technical team needs to ensure that the relevant findings are reported somewhere in the framework so that they can be taken into account in decision-making.

Because qualitative evidence is often broad in nature, it may be relevant to more than one of the frameworks included in a guideline. Additionally, findings from several QES may be relevant to one or more frameworks. For example, a QES conducted for forthcoming WHO guidance on communication interventions to inform and educate caregivers on routine childhood vaccination in the African Region included a broad finding that the acceptability of vaccination communication interventions appears to be influenced by several factors, including people’s trust in and relationship with the information source as well as the manner in which the information is presented [27]. This finding was judged by the technical team to be applicable across all of the communication interventions included in the guidance. Such findings can either be repeated in each relevant framework or included in an overarching text linked to multiple frameworks. For example, in the WHO antenatal care guideline, the evidence on how people value the outcomes was found to be similar across groups of interventions. The technical team therefore summarised this evidence in a separate overarching narrative rather than repeating the same information in each framework [11].

Another reason to use an overarching or cross-cutting approach is that it can be challenging to summarise qualitative evidence succinctly without losing meaning and data on context. Where an overarching narrative is developed, the technical team need to ensure that it is clear to the guideline panel that the qualitative evidence for several frameworks is presented in an overarching document, and each EtD needs to link to this document. Importantly, whilst the qualitative evidence might be the same for different guideline questions, the guideline panel’s judgements for each criterion might differ, depending on the intervention evaluated in each question.

Wider, less specific findings may need to be used in relation to an intervention where more specific findings are not available. For instance, a finding may be available regarding people’s views of receiving health messages via mobile phones but not on people’s views regarding such messaging for the particular health issue that is the focus of the guideline question.

Qualitative evidence may have direct relevance to a guideline question or may be indirectly relevant. Indirect evidence, for example, qualitative evidence regarding a related intervention or context to the one of interest, can be included in the ‘Research evidence’ section of the EtD framework. However, it may be helpful to indicate clearly to users, for instance, through the CERQual assessment of confidence, that the evidence is indirectly relevant.

Overall, the technical team needs to ensure, firstly, that each framework includes sufficient information to inform a recommendation and, secondly, that people using the recommendations are able to understand the justification for each recommendation from the evidence presented.

#### Weave the individual QES findings into a narrative for each framework criterion

Once the findings have been allocated to a specific criterion, the guideline technical team needs to weave these findings into a single, short narrative for inclusion in an EtD framework. This narrative should also include the CERQual assessments for the included findings. In our experience, it is often the case that several synthesis findings, from one or more QES, are relevant to a single framework criterion.

We do not have evidence on the optimal length of the narrative text for framework criteria and this is influenced by the nature of the findings and the number of frameworks that a guideline panel has to consider as part of a guideline process. However, the following principles may be helpful:

- The narrative should include the key points from the findings that are relevant to the decision that the framework will inform.
- The narrative should include enough information on the context of the findings (for instance, that participants were from remote rural communities) to reduce ambiguity and allow interpretation, including of the relevance of the evidence as assessed using CERQual.
- A graded entry or layered approach to presenting information may be helpful [28, 29], with the most summarised information presented in the EtD framework. In a graded entry format, users can then navigate from this summary to more detailed information, for example, the full summary of qualitative findings table, and from there to the full synthesis report. An example of this is available here: www.optimizemnh.org.
- Users should be able to trace back from the narrative to the individual findings that informed the narrative. Traceability can be enhanced by giving a unique code to each QES finding and including these codes in the narrative.

As technical team members, we have found that the narrative summarising relevant QES findings usually needs several iterations before it is finalised for inclusion in an EtD. Tables 2, 3, 4, 5 and 6, and Additional files 2, 3 and 4, show examples of how multiple synthesis findings may contribute to a narrative summary in an EtD framework.

**Table 2:** Example of using qualitative evidence to populate the evidence-to-decision framework criterion on how people value the outcomes

Guideline and framework	Source of the findings	Qualitative evidence synthesis findings	Text developed from these finding/s for the values criterion of the framework/s
Antenatal care (ANC) guideline – nutritional intervention frameworks [11]	Commissioned synthesis [35]	Synthesis Finding 10 – Brief and cursory encounters with healthcare providers during ANC appointments were highlighted by a number of women in a variety of contexts. The impersonal nature of the ANC encounter, coupled with a reliance on tests and procedures rather than conversation, left women feeling isolated and disenfranchised Synthesis Finding 11 – Women’s willingness to engage with ANC was enhanced when healthcare providers were perceived to be authentic and kind. A friendly, respectful and attentive approach was appreciated by women, especially those who were feeling worried or anxious about their pregnancy Synthesis Finding 23 – In many countries, women visit ANC providers to acquire knowledge and information about their pregnancy and birth. In situations where this is provided in a useful, appropriate and culturally sensitive manner, sometimes through the use of pictures and stories, it can generate a sense of empowerment and acts as a facilitator to further engagement. In situations where this approach is not adopted, e.g. where tests are not explained properly or information is infused with medical jargon or is outdated and irrelevant, it acts as a barrier and limits further access	A scoping review of what women want from ANC informed the outcomes for the ANC guideline. Evidence showed that women from various resource settings valued having a positive pregnancy experience comprising three equally important components, namely effective clinical practices (interventions and tests, including nutritional supplements), relevant and timely information (including dietary and nutritional advice), and psychosocial and emotional support, provided by knowledgeable, supportive and respectful healthcare practitioners to optimise maternal and newborn health (high confidence in the evidence)

**Table 3:** Example of using qualitative evidence to populate the evidence-to-decision framework criterion on gender, health equity and human rights impacts – ‘direct’ equity impacts

Guideline and framework	Source of the findings	Qualitative evidence synthesis findings	Text developed from these finding/s for the equity criterion of the framework^a^
Communication interventions to inform and educate caregivers on routine childhood vaccination in the African Region – Face-to-face interventions and community-aimed interventions (World Health Organization Regional Office for Africa: Guidance on Communication Interventions to Inform and Educate Caregivers on Routine Childhood Vaccination in the African Region, forthcoming)	Existing synthesis [27]	Synthesis finding 6 – Parents who had migrated to a new country had difficulty negotiating the new health system and accessing and understanding vaccination information (low confidence in the evidence) Synthesis finding 16 – Parents felt that the vaccination card was a potentially important source of vaccination information, for instance, about the names of the diseases, the names of the vaccines and the date for the next appointment. However, some parents and informal caregivers found it difficult to read and understand this information (moderate confidence in the evidence) Synthesis finding 32 – Parents wanted information that was presented in an understandable way that avoided technical terms and jargon to facilitate their assessment of the content. Parents sometimes found medical terminology used in medical research or by their healthcare provider difficult to understand and evaluate. Misunderstanding and lack of access were further compounded when written information was presented to illiterate mothers, when the mother’s education level was not taken into account when providing information, or when health workers did not provide any information at all. Parents also wanted information communicated in a language that they could understand. Some parents also found presentations in the media unclear due to the mixing of anecdotal and scientific evidence to create an impression of balance. A clear presentation of information was important for parents to feel like they had understood the information they had received (moderate confidence in the evidence)	Certain circumstances may make it particularly difficult for people to understand vaccination information. These include: • literacy level: parents who are illiterate or who have lower levels of education may find information difficult to access, particularly when information is presented in writing or includes technical terms and jargon • unfamiliarity with the health system: parents who have migrated to a new country may have insufficient knowledge about how immunisation services and policies work in their new countries concerning, for example, schedules and appointments • language: parents who speak languages other than those most commonly spoken within the health services or the setting in which they live may find information difficult to access

^a^The text has been adapted from the original guideline for the purposes of these examples

**Table 4:** Examples of using qualitative evidence to populate the evidence-to-decision framework criterion on gender, health equity and human rights impacts – ‘indirect’ equity impacts

Guideline and framework	Source of the findings	Qualitative evidence synthesis summary findings	Text developed from these finding/s for the equity criterion of the framework
Communication interventions to inform and educate caregivers on routine childhood vaccination in the African Region – Face-to-face interventions and community-aimed interventions (World Health Organization Regional Office for Africa: Guidance on Communication Interventions to Inform and Educate Caregivers on Routine Childhood Vaccination in the African Region, forthcoming)	Existing synthesis [27]	Synthesis finding 13 – Health workers are an important source of vaccination information for parents (high confidence in the evidence) Synthesis finding 25 – Some parents distrusted or lacked confidence in information sources linked to the government. They considered these to be biased, to be withholding information or to be motivated by financial gain (moderate confidence) Synthesis finding 36 – Parental misconceptions about vaccination were sometimes based on information that they had received from health workers (moderate confidence in the evidence)	Issues hypothesised from the evidence: • The evidence shows that health workers are an important source of vaccination information for most parents. We can assume that population groups with poor access to health workers will also have less access to vaccination information. In addition, we can assume that the problem of vaccination misinformation from health workers is likely to be more common for people living in areas where it is difficult to recruit and retain well-trained health workers. • The evidence shows that some parents distrust or lack confidence in information sources linked to the government. Where population groups have low levels of trust in the government, for instance, because of political tensions or ethnic conflict, we can assume that they may find it particularly difficult to trust information from government healthcare providers

**Table 5:** Example of using qualitative evidence to populate the evidence-to-decision framework criterion on the acceptability of the intervention

Guideline and framework	Source of the findings	Qualitative evidence synthesis findings	Text developed from these finding/s for the acceptability criterion of the framework
Intrapartum care guideline – episiotomy [13]	Commissioned synthesis (women’s findings) [45]	Synthesis finding 1 - Subordination and compliance (high confidence). In a number of contexts, women handed over responsibility for their care to providers – sometimes this was done voluntarily but, more often, choices or decisions were taken out of their hands. Women were not asked for consent for certain procedures (e.g. episiotomy) or were coerced or bullied into having interventions against their will. Synthesis finding 2 - Perception of pain (moderate confidence). Some women found this procedure extremely painful. In certain situations, the procedure was performed without anaesthetic and was described as being worse than the pain associated with childbirth. For others, particularly those with previous experience of episiotomy, the pain was tolerable Synthesis finding 3 - Lack of respect (low confidence). In a number of instances, women were not given any choice about having an episiotomy. Their views and concerns were disregarded by health professionals, they were not asked for consent and, in some cases, were not given any anaesthesia to ease the pain Synthesis finding 4 - [Episiotomy facilitates] an easier birth (low confidence). Amongst some women there was a belief that the use of episiotomy helped to make birth easier by reducing the length of labour and the level of pain Synthesis finding 5 - Pre-procedure anxiety (low confidence). Some women were worried about the implications of having an episiotomy and felt anxious about potential effects on their body image or their bodily functions Synthesis finding 6 - Post-procedure discomfort (moderate confidence). Some women experienced both short- and long-term discomfort following an episiotomy. In the short term, this involved difficulty sitting down, using the toilet or having sex and, in the longer term, women experienced general perineal pain up to 18 months after surgery	In a qualitative systematic review exploring women’s and providers’ views and experiences of intrapartum care, women felt they were poorly informed about the reasons for performing an episiotomy and were rarely asked for their permission (high confidence in the evidence). Review findings suggest that women preferred to minimise the level of pain experienced from cutting and stitching, as well as the levels of discomfort experienced following episiotomy (high confidence in the evidence). In addition, they may be ill-prepared for the pain associated with the procedure or the potential short- and long-term consequences (perineal discomfort, difficulty performing normal day-to-day activities, aesthetic deformities, effect on sex life) (low confidence in the evidence). In some instances, women felt that their concerns were ignored or dismissed by staff, whom they perceived to be rude and insensitive (low confidence in the evidence). The review findings also suggest that, in certain countries (e.g. Brazil), women might hold the belief that an episiotomy facilitates a smoother birth (shorter labour, less pain) (low confidence in the evidence). This may be based on an established cultural acceptance of the procedure, largely generated by healthcare providers (low confidence in the evidence)

**Table 6:** Examples of using qualitative evidence to populate the evidence-to-decision framework criterion on the feasibility of the intervention

Guideline and framework	Source of the findings	Qualitative evidence synthesis findings	Text developed from these finding/s for the feasibility criterion of the framework
Antenatal care (ANC) guideline – group ANC [11]	Commissioned synthesis (provider findings) [35]	Synthesis finding 1 - Continuity of care (moderate confidence). Providers offering group ANC felt that the model gave them the opportunity to practice continuity of care and this was seen as a facilitator for the delivery of good quality ANC. Where providers were not able to offer continuity of care, this was viewed as a barrier to the delivery of quality ANC Synthesis finding 2 - Condition of clinic (moderate confidence). Providers in sub-Saharan Africa feel that clinics are in a very poor condition and are not amenable to the delivery of ANC. They cited a lack of running water or electricity, no phone lines and dirty rooms as specific concerns	Evidence from high resource settings suggests that health professionals view the facilitative components of group antenatal care as a skill requiring additional investment in terms of training and provider commitment (moderate confidence in the evidence). Some providers also feel that clinics need to be better equipped to deliver group sessions, i.e. clinics need to have large enough rooms with adequate seating (moderate confidence in the evidence)
ANC guideline – midwife-led continuity of care [11]	Commissioned synthesis (provider findings) [35]	1. Staff shortages (high confidence). Providers felt that their ability to deliver high quality ANC was restricted by a shortage of frontline staff	Qualitative evidence from a variety of resource settings highlights concerns among providers about potential staffing issues, e.g. for the delivery of case-load or one-to-one approaches (high confidence in the evidence)
Intrapartum care guideline – episiotomy [13]	Commissioned synthesis (provider findings) [45]	Synthesis Finding 3 – Some health professionals were reluctant to change their practice of routine episiotomy because of entrenched views based on experience and opinion rather than evidence. Midwives felt powerless to change practice because of patriarchal and hierarchical systems resistant to change Synthesis Finding 5 – Some health professionals performed episiotomy in certain situations (baby too big, tight perineum, preventing a tear, fetal bradycardia, non-reassuring fetal status, shoulder dystocia) and cited a lack of hospital policy and limited access to current evidence as mitigating factors Synthesis Finding 6 – In some contexts, health professionals felt that an episiotomy enabled them to ‘manage’ labour and birth. In a clinical sense, they felt an episiotomy limited the potential for tearing and, from a workload perspective, helped to speed up a slow labour and ease bed space pressures	Information from a qualitative systematic review exploring women’s and providers' views of intrapartum care suggest that a practice of selective/restrictive episiotomy would be easier to implement, especially in settings where resources may be limited (high confidence in the evidence). However, in certain contexts, staff may have limited access to current research evidence (because of resource constraints) and subsequently have no clear policies or protocols to guide practice in this area (high confidence in the evidence). As a result, clinical practice is based on established, hierarchical, unwritten ‘rules’ and/or competence in performing the procedure (high confidence in the evidence)

#### Consider whether any additional considerations need to be included in each framework

There may be circumstances in which other qualitative, or related, evidence or information needs to be included for a particular framework criterion, in addition to the findings of the contributing QES. This additional information may also be needed where no relevant evidence was found by the QES. This additional evidence might include [30]:

- Descriptions of conceptual or theoretical frameworks that help in understanding the QES findings or that place these within a wider context
- Findings from individual qualitative studies that provide important contextual information related to the setting of the recommendation or decision but were not eligible for inclusion in the QES
- Plausible reasons for anticipating that the intervention might or might not be acceptable to key stakeholders or might be difficult to implement, particularly where little or no evidence on acceptability or feasibility was found for an intervention
- Any assumptions made in relation to the findings presented and, if relevant, the basis for those assumptions

This additional evidence or information can be included in the ‘Additional considerations’ section for the relevant framework criterion.

### Populating evidence-to-decision framework criteria with qualitative evidence – examples in relation to relevant framework criteria

Here, we describe in more detail how we have used qualitative evidence to identify issues relevant to specific criteria within the EtD framework and present examples of the approaches we have used.

To ensure that all relevant stakeholders and contexts are considered in a QES commissioned for a guideline, it is helpful at the scoping stage for the guideline panel to consider which stakeholders and contexts are most important. In doing so, they should take into account the anticipated coverage of the guideline (for example, is it intended for a specific country, or is it intended to provide global recommendations?) and those affected both directly and indirectly by the guideline (for instance, those affected indirectly may include the partners of women receiving an intervention). This is discussed further in paper 1 in this series [16].

#### How people value the outcomes

The guidance on populating an EtD framework notes that the direction of a recommendation may change where there is uncertainty about how those affected by this intervention value the outcomes of interest. Additionally, the strength of a recommendation may be affected by research evidence showing that different groups value the desirable and undesirable effects differently [30].

There are at least three complementary sources for evidence on how people value outcomes in relation to an intervention or option, namely studies that have measured utility values – a measure of how strong people’s preference is for a specific health state; studies that “*directly measure the choices people make when presented the probabilities of the desirable and undesirable effects, a description of those outcomes (health states) and information about when they would occur and how long they would last*” ([30] p. 18); and qualitative evidence from studies that explore people’s views of the impacts of different health issues and interventions.

To date, we have limited experience in using qualitative evidence to understand how people value the outcomes of interest for a guideline. In the WHO antenatal care guideline, a QES done at the start of the process helped the guideline technical team identify that a ‘positive pregnancy experience’ was highly valued by women. This included “*maintaining physical and sociocultural normality; maintaining a healthy pregnancy for mother and baby; effective transition to positive labour and birth; and achieving positive motherhood*” ([31] p. 532). The guideline technical team translated this finding into a framework of actionable components that could achieve this desirable outcome. These components included psychosocial and emotional support, relevant and timely information, and effective clinical practices. To populate the framework criterion on how people value the main outcomes, the technical team initially prepared qualitative findings statements tailored to the different groups of questions in the antenatal care guideline, such as nutritional interventions and maternal assessment. For guidelines that include only a small number of related interventions, a single ‘values’ statement could be sufficient for all of the guideline frameworks. Table 2 and Additional file 2 provide examples from two guidelines on how findings from qualitative evidence syntheses were used to address this criterion in EtD frameworks.

#### Gender, health equity and human rights impacts

The guidance on populating an EtD framework notes that technical teams “*should evaluate potential impacts on equity in relation to specific characteristics that are likely to be associated with disadvantage in relation to the question they are addressing*” ([30] p. 23). There are two ways in which we, as guideline technical teams, have used qualitative evidence to populate the gender, health equity and human rights impacts section within the EtD framework; firstly, issues may be identified directly from the findings of a QES. In these cases, we simply summarise these data for this criterion of the framework. Table 3 and Additional file 3 show examples from two WHO guidelines of how, as the technical team, we moved from qualitative evidence synthesis findings to a narrative text for the gender, equity and human rights criterion.

Secondly, where a QES undertaken for a guideline does not identify gender, health equity or human rights issues explicitly, it may be possible to infer these from the findings through discussion within the technical team or experts in the field. A narrative summary of the issues can then be created (Table 4). Where this is done, it is important to indicate to those making recommendations that these issues were hypothesised from the evidence rather than being described there explicitly and the technical team should consider including these issues under ‘Additional considerations’ in the EtD framework.

#### Acceptability and feasibility

We have defined acceptability as “*the extent to which that intervention is considered to be reasonable among those receiving, delivering or affected by the intervention*” ([7] p. 186). The feasibility of an intervention can be seen as “*the likelihood that it can be properly carried out or implemented in a given context*” ([7] p. 187). An intervention may be more or less acceptable and feasible to different stakeholders in different contexts.

In our experience, qualitative evidence on the acceptability and feasibility of different interventions is often linked. For example, when an intervention involves additional costs for service users, it may be associated with both lower acceptability and lower feasibility. The technical team will often need to take pragmatic decisions on whether to report QES findings in the acceptability or feasibility sections of the EtD framework. As a recommendation is based on judgements regarding all of the evidence presented in a framework, where best to place a specific relevant QES finding is less important than ensuring it is included.

Our experience has also highlighted that qualitative studies often do not include in-depth data on intervention feasibility. This may be because these studies often focus on the views of service users or providers regarding a health issue, and do not include the views of healthcare managers or explore factors affecting the governance or financing of interventions or programmes [32]. This evidence gap has led us to carry out multi-country case studies for several guidelines. These included a broader set of information sources, including programme descriptions and mixed method programme evaluations, that might provide evidence on factors influencing the feasibility and implementation of an intervention [33, 34] (Muloliwa AM, Cartier Y, Ames H, Oku A, Bosch-Capblanch X, Cliff J, Glenton C, Hill S, Kaufman J, Oyo-Ita A, et al; Synthesis of health systems barriers and facilitators to scaling up vaccination communication interventions in Cameroon, Nigeria, and Mozambique, in preparation). However, we found that these wider sources provided less data than anticipated as, firstly, we found fewer programme descriptions and evaluations than we expected and, secondly, those that we found generally included only very thin data. These experiences suggest that it may be more useful to collect additional data on the feasibility of guideline interventions through qualitative key informant interviews with programme managers and decision-makers. These data can then be either incorporated into the relevant QES or reported separately in the EtD framework. Interview studies should be planned at the same time as the evidence synthesis protocols for a guideline are being developed.

When the technical team starts to develop the summary narratives for the acceptability and feasibility sections of the EtD framework, they should also consider how to convey the extent to which the evidence shows similarities and differences across stakeholders and contexts. In some cases, it may be appropriate to include separate narratives for different stakeholders or contexts. When no specific qualitative evidence for a particular option, stakeholder group or context is found, it may be possible for the technical team to draw inferences from findings for other options, stakeholders or contexts. For instance, findings on the acceptability to mothers of childhood vaccination communication interventions may also apply to other caregivers. Where inferences are made, this should be made clear in the relevant framework.

Tables 5 and 6 and Additional file 4 include examples from two guidelines of how we, as the guideline technical team, moved from summary QES findings to a narrative summary of acceptability and feasibility for an EtD framework. Additional file 4 also includes an example of where no specific evidence on the acceptability of an option was found, and inferences from other QES findings were used.

### How qualitative evidence synthesis findings may influence guideline recommendations

When making a recommendation, a guideline panel should take into account all of the evidence presented in the EtD framework. The extent to which the qualitative evidence included in a framework influences or drives a decision regarding a particular recommendation will vary across the questions considered by a guideline – in some cases, a decision may be driven by other information presented in the framework. Regardless, all judgements should be supported by a clear justification that refers to the key criteria that drove the decision.

Two examples from recent guidelines illustrate how qualitative evidence can inform decisions in different ways. The first example is from the WHO antenatal care guideline [11] and concerns recommendations on the relief of physiological symptoms in pregnancy. A QES conducted for the guideline identified, in relation to acceptability, that pregnant women in LMICs are more likely to turn to traditional or alternative healers, herbal remedies or traditional birth attendants to treat physiological symptoms (moderate confidence in the evidence) and that they are less likely to engage with health services if their beliefs, traditions and socioeconomic circumstances are ignored or overlooked (high confidence in the evidence) [35]. Further qualitative findings indicated that a lack of suitably trained staff could limit the feasibility of certain interventions, such as acupuncture, for relief of physiological symptoms (high confidence in the evidence). These findings led to most of the interventions for physiological symptoms being recommended, but the recommendations specifically note that use should be based on a woman’s preferences and available options.

The second example is from the WHO guideline on digital interventions for health systems strengthening [18] and concerns a recommendation on the use of targeted client communication via mobile devices for behaviour change related to sexual, reproductive, maternal, newborn, child and adolescent health. The effectiveness evidence suggested that this intervention may have positive impacts on some behaviours and health outcomes relating to modern contraception use by adults, adherence to antiretroviral medications, antenatal care, skilled birth attendance and childhood vaccinations. However, the evidence also indicated that the intervention may make little or no difference to other outcomes and has some unintended negative consequences. A QES conducted for the guideline indicated that targeted client communication is generally acceptable, but that some population subgroups, particularly vulnerable populations, have concerns about the confidentiality of health information, particularly for sensitive health issues. Additionally, access to and use of communication via mobile devices may be particularly difficult for certain groups of people such as those with low literacy levels (Ames HMR, Glenton C, Lewin S, Tamrat T, Akama E, Leon N; Clients' perceptions and experiences of targeted digital communication accessible via mobile devices for reproductive, maternal, newborn, child and adolescent health: A qualitative evidence synthesis, forthcoming). The guideline panel decided to recommend targeted communication via mobile for behaviour change regarding sexual, reproductive, maternal, newborn and child health, but with the condition that concerns about sensitive content and data confidentiality are adequately addressed [18]. A further example is provided in Additional file 5.

At present, we have limited knowledge of how best to integrate different types of evidence within EtD frameworks and how to present these different types of evidence to guideline panels. We also do not yet have a good understanding of how guideline panels use and adjudicate different types of evidence (quantitative, qualitative) addressing different types of questions (effectiveness, feasibility, etc.) in making a decision. Further research is needed in these areas [36–39]. The lessons we have learnt suggest that it may be helpful to:

- Provide guideline panels, in advance of their meeting, with information on the purpose of the EtD framework; the criteria within it; the types of evidence that will be used to address each criterion, including how qualitative evidence will be used; what constitutes research evidence and what additional information can be considered; and how the decision-making process will work [30]. This can be done through online webinars or written information
- Reiterate this information at the start of the guideline panel meeting
- Present the different types of evidence as clearly and succinctly as possible; using an EtD framework assists with this
- Prompt guideline panels to justify their recommendations in relation to the full body of evidence, including the qualitative evidence, in an EtD framework

## Discussion

As members of the technical teams for a series of WHO guidelines, we used QES as our main source of information to populate specific EtD framework criteria, and this paper reflects the lessons we have learnt to date. As Graham et al. [40] noted some time ago in their knowledge-to-action cycle, knowledge creation involves moving from a process of identifying knowledge – for instance, from primary research studies – to critically appraising that knowledge, incorporating it into an evidence synthesis and then using the evidence synthesis findings in derivative tools or products. In this paper, we have described this process in relation to the use of qualitative evidence to support guideline development, showing how findings from QES can be translated into summary formats that can then feed into an EtD framework.

This paper is based on our experience of using QES findings within WHO guideline development. In drawing out the lessons we have learnt, we have tried to ensure that these could be applied across a wide range of health guidelines. The approaches we describe may also be useful for decision-making processes in other sectors that aim to utilise qualitative evidence. However, we acknowledge that our experiences are limited to the WHO context and the range of guidelines in which we have been involved, and also to the EtD framework approach. As we note below, further work is needed to explore the application of the learnings described in this paper to guidelines in other areas. Further research on how qualitative evidence is understood and used within decision-making processes may also lead to insights that enhance and extend the guidance outlined in this paper.

**Fig. 3:**
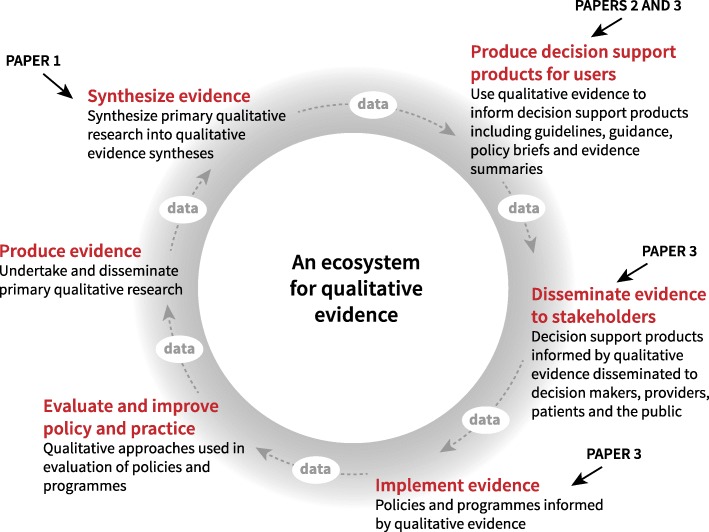
How this series of papers contributes to strengthening the ecosystem for qualitative evidence. Adapted from http://magicproject.org/research-and-tools/the-evidence-ecosystem/

Synthesising evidence and producing guidance are just two elements of what has been termed the ‘evidence ecosystem’ [41–43] (Fig. 3). As we have noted elsewhere, recent advances within the field of qualitative research mean that we now have in place most of the parts of an ecosystem for qualitative evidence [8]. As this ecosystem is strengthened, more qualitative evidence will become available to help address the questions that stakeholders identify when making decisions about the use of health interventions [44].

Below we identify a number of research questions, along with implications for practice, for those working on guideline development.

### Implications for practice

- Guideline technical teams ideally need to include, or have access to, people with skills in QES, GRADE-CERQual and in populating and using EtD frameworks. This has implications for the resources required to undertake a guideline development process
- The scoping phase of guideline development is critical for identifying the interventions, stakeholders and contexts relevant to the guideline questions. Decisions on these aspects will shape the scope of the QES undertaken for the guideline and adequate time needs to be allowed for this process, including for interactions with the QES teams
- Technical teams should be aware that the findings of scoping and other QES conducted for a guideline may impact on the range and scope of effectiveness reviews for the guideline. QES findings regarding which interventions are seen as important by stakeholders and how people value different outcomes may need to be fed back into the scoping process for effectiveness reviews commissioned for a guideline
- As the number of published QES increases, it is more likely that an existing QES may be found that addresses some or all of the guideline questions. Searches for existing QES should be done before a new QES is commissioned
- A technical team may need to commission both broad QES that cover multiple guideline interventions as well as ‘mini-QES’ that focus on one specific intervention. It can sometimes be useful to use rapidly conducted ‘mini-QES’ to address important gaps in the evidence available for a guideline
- Close collaboration between the QES authors and the guideline technical team responsible for populating the EtD framework may help to ensure that the QES findings are developed and tailored to each EtD framework, and relevant criteria within these frameworks. Close collaboration may also help to ensure congruence between the findings in the published QES and those included in the frameworks
- Users of EtD frameworks need to be able to easily identify the sources of qualitative and other evidence presented in a framework. This traceability requires careful attention to documenting how evidence moves from primary studies, to a QES, and then into a framework
- Technical teams should consider the information and training needs of groups making recommendations in relation to qualitative evidence, and in the use of this evidence in guidelines. Information sessions or training for these groups may be needed in advance of formal meetings of these groups

### Implications for research

- As the number of reviews that include both qualitative and other kinds of data increase (so-called mixed-method reviews), research will be needed on strategies for including findings that are based on multiple types of data in frameworks, and how to assess how much confidence to place in these findings. Mixed method approaches may be particularly relevant to the ‘values’ and ‘acceptability’ criteria within the EtD framework as survey data on these issues are sometimes available
- In populating an EtD framework, a technical team has to strike a balance between informativeness and length. To keep EtD frameworks to a manageable length, we have typically used summarised QES findings in these documents and then referred guideline panel members to the relevant Summary of Qualitative Findings for further detail. Future research needs to explore guideline panel members’ views on the level of detail they find useful in an EtD framework and their experience of graded entry formats to present information for decision-making
- Future research should consider the circumstances in which it might be appropriate to use findings from individual qualitative studies in an EtD framework
- Future research should explore guideline panel’s preferences regarding different ways of presenting qualitative evidence that cuts across several guideline questions, and therefore frameworks
- Future research should explore how guideline panels understand, use and adjudicate the different types of evidence that may be included in EtD frameworks, including qualitative evidence, and the roles of the technical team in prompting these groups to take account of qualitative evidence during their deliberations
- Future research needs to explore the application of the learnings described in this paper to guidelines in other areas such as social care and education

## Conclusion

This paper explores how QES findings can be used to populate key evidence to decision framework criteria in the context of guideline development. We have demonstrated the value of investing in QES as part of a guideline development process, and the roles that qualitative evidence can play in representing the views and experiences of stakeholders [8]. We have also identified a number of issues that deserve further exploration, and look forward to seeing a growing body of research and experience in these areas.

Box 1 Commissioning a qualitative evidence synthesis (QES) to inform a guideline Stages:

- Identifying the areas and topics for a QES – the guideline technical team identifies the broad areas or topics for which a QES will be needed; this could include a QES to inform the scoping of the guideline or a QES to inform specific criteria that are part of an evidence-to-decision (EtD) framework (such as the acceptability and feasibility of an intervention)
- Identify synthesis leads and teams – ideally teams should include at least one person with extensive experience in qualitative evidence synthesis and a person with content area expertise in relation to the guideline topic
- Discussion of the scope of each synthesis – where more than one synthesis is being commissioned for a guideline, it may be helpful to hold a meeting of the guideline technical team and the synthesis lead authors to consider the scope and objectives of each synthesis. This discussion should include the range of questions that the synthesis will consider, in relation to the EtD criteria used for the guideline. For example, should the synthesis consider equity and human rights issues and resource use issues, in addition to intervention acceptability and feasibility? The discussion should also cover which synthesis approach/es to use, based on which would be most appropriate for addressing the synthesis objectives, how the QES findings will be used within the EtD frameworks, and how best to tailor the synthesis to address the specific needs of a guideline process
- Preparing the terms of reference – this would include which databases will be searched; how the synthesis findings will be prepared for the guideline, including the types of information and data that will be included in the CERQual Qualitative Evidence Profiles and Summary of Qualitative Findings tables; how an assessment of confidence in the evidence will be made; the content of the final manuscript; and how the technical team and synthesis leads will communicate during the process of producing the syntheses
- Develop a protocol for each synthesis – where more than one synthesis is commissioned for a guideline, it may be helpful to ensure (as far as possible) that the synthesis processes are standardised across protocols and make sense in relation to the synthesis objectives. Where possible, the protocol/s should be made publicly available (through, for example, registering the synthesis with Cochrane EPOC, Prospero etc.)

A budget for the review should be estimated. In addition to time to conduct the review, person-time needs be included for undertaking a CERQual assessment; several rounds of discussion of the review findings between the synthesis team and the guideline technical team, to ensure that the findings are written as clearly as possible and are congruent with the underlying data; reviewing any summarised findings prepared for different domains of the EtD frameworks; and preparing the synthesis for publication

A qualitative evidence synthesis is labour intensive process and the additional stages needed to prepare the findings for a guideline process generally add additional person-time to the process

## Additional files

Additional file 1: Example of a GRADE evidence-to-decision framework. (DOCX 206 kb)

Additional file 2: Example of using qualitative evidence to populate the evidence-to-decision framework criterion on how people value the outcomes. (DOCX 16 kb)

Additional file 3: Example of using qualitative evidence to populate the evidence-to-decision framework criterion on gender, health equity and human rights impacts – ‘direct’ equity impacts. (DOCX 16 kb)

Additional file 4: Examples of using qualitative evidence to populate the evidence-to-decision framework criterion on the acceptability of the intervention. (DOCX 21 kb)

Additional file 5: How qualitative evidence has influenced the formulation of recommendations – example from the WHO antenatal care guideline. (DOCX 16 kb)

## Data Availability

Not applicable.

## Abbreviations

EtD: evidence-to-decision
GRADE: Grading of Recommendations, Assessment, Development and Evaluation
GRADE-CERQual: Grading of Recommendations, Assessment, Development and evaluation - Confidence in the Evidence from Reviews of Qualitative Research
QES: qualitative evidence synthesis
SoF: summary of findings
