# Supplementary figures and images for: Qualitative Evidence Synthesis (QES) for Guidelines: Paper 2 – Using qualitative evidence synthesis findings to inform evidence-to-decision frameworks and recommendations
Source: Health Res Policy Syst. 2019 Aug 8;17:75. doi: 10.1186/s12961-019-0468-4 (PMC6686513; doi:10.1186/s12961-019-0468-4)

**Additional file 1: Example of a GRADE evidence-to-decision framework**


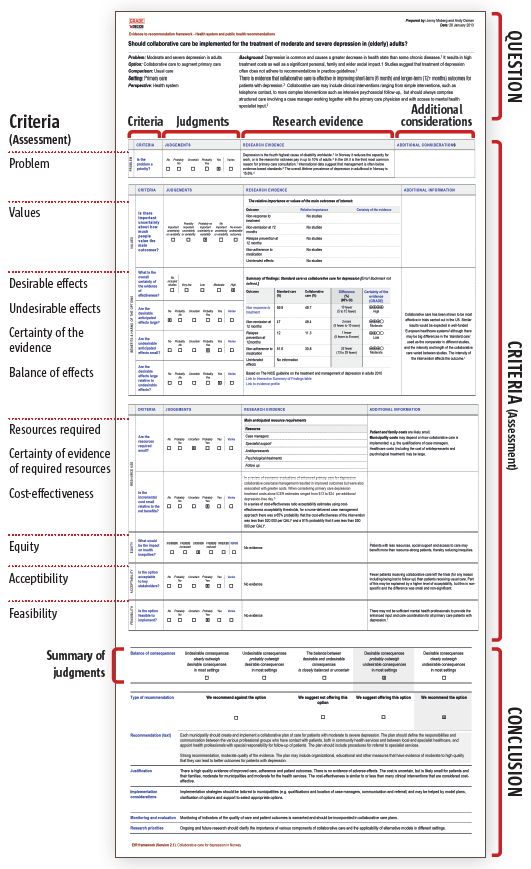

Supplement: Supplementary file 1 — Example of a GRADE evidence-to-decision framework. (DOCX 206 kb) [file 12961_2019_468_MOESM1_ESM.docx]
