# Supplementary material for: Qualitative Evidence Synthesis (QES) for Guidelines: Paper 2 – Using qualitative evidence synthesis findings to inform evidence-to-decision frameworks and recommendations
Source: Health Res Policy Syst. 2019 Aug 8;17:75. doi: 10.1186/s12961-019-0468-4 (PMC6686513; doi:10.1186/s12961-019-0468-4)
Supplement: Supplementary file 2 — Example of using qualitative evidence to populate the evidence-to-decision framework criterion on how people value the outcomes. (DOCX 16 kb) [file 12961_2019_468_MOESM2_ESM.docx]

**Additional file 2: Example of using qualitative evidence to populate the evidence-to-decision framework criterion on how people value the outcomes**

| **Guideline and framework** | **Source of the findings** | **Qualitative evidence synthesis findings** | **Text developed from these finding/s for the values criterion of the framework/s** |
| --- | --- | --- | --- |
| Intrapartum care guideline / pain relief frameworks [1] | Commissioned synthesis [2] | Synthesis Finding 38 - Women wanted to know what was going on during their labour and welcomed any information about progress or clinical practices, particularly if this was delivered by health-professionals in a jargon-free manner. Where their requests for information were dismissed or ignored women felt frustrated, resentful or disempowered.  Synthesis Finding 40 - Women were apprehensive about labour and birth and highlighted their concerns in a variety of ways including fears about pain, of having certain interventions (caesarean section and episiotomy), of giving birth to an unhealthy baby, of vaginal examinations, of complications and, ultimately, of death.  Synthesis Finding 46 – Women described feeling reassured by the availability of pain relieving medications, regardless of whether they chose to use them. For some, the use of analgesia (particularly epidural) provided immediate relief from pain and made birth more manageable, while for others, pain relief (using epidural) proved ineffective. | In a review of qualitative studies looking at what women want from intrapartum care, findings suggest that most women, especially those giving birth for the first time, are apprehensive about childbirth (high confidence in the evidence), and in certain contexts and/or situations may welcome interventions that provide relief from pain (low confidence in the evidence). Where such interventions are being considered, women would like to be informed about the nature of the intervention and, where possible, given a choice (high confidence in the evidence). |

**References**

1. WHO: **WHO recommendations: intrapartum care for a positive childbirth experience**. Geneva, Switzerland: World Health Organization; 2018.

2. Downe S, Finlayson K, Thomson G, Hall-Moran V, Feeley C, Oladapo OT: **WHO recommendations for interventions during labour and birth: Qualitative evidence synthesis of the views and experiences of service users and providers**. 2018 (Unpublished).
