# Supplementary material for: Qualitative Evidence Synthesis (QES) for Guidelines: Paper 2 – Using qualitative evidence synthesis findings to inform evidence-to-decision frameworks and recommendations
Source: Health Res Policy Syst. 2019 Aug 8;17:75. doi: 10.1186/s12961-019-0468-4 (PMC6686513; doi:10.1186/s12961-019-0468-4)
Supplement: Supplementary file 3 — Example of using qualitative evidence to populate the evidence-to-decision framework criterion on gender, health equity and human rights impacts – ‘direct’ equity impacts. (DOCX 16 kb) [file 12961_2019_468_MOESM3_ESM.docx]

**Additional file 3: Example of using qualitative evidence to populate the evidence-to-decision framework criterion on gender, health equity and human rights impacts – ‘direct’ equity impacts**

| **Guideline and framework** | **Source of the findings** | **Qualitative evidence synthesis findings** | **Text developed from these finding/s for the equity criterion of the framework*** |
| --- | --- | --- | --- |
| Intrapartum care guideline/episiotomy [1] | Existing synthesis [2] | Synthesis finding 8: ‘Across multiple contexts, women referred to a “fear of  cutting” as a deterrent to pursuing facility delivery. Women who mentioned a fear of cutting usually did not differentiate between episiotomy and a caesarean section; rather, they referred to any form of perineal or abdominal incision as  “cutting”. Women feared cutting due to perceived longer hospital stays, higher cost, perceived unjustified operation, social stigma, and potential problems with future sexual relations.’ (moderate confidence in the evidence) | Qualitative evidence from a review of barriers and facilitators to facility-based birth indicates that many women have a “fear of cutting” by health workers and this is probably a significant barrier to the uptake of facility-based birth by women in LMICs. Therefore, reducing episiotomies might have a positive impact on health equity by increasing facility-based birth coverage among women in LMICs. |

* The text has been adapted from the original guideline for the purposes of these examples

**References**

1. WHO: **WHO recommendations: intrapartum care for a positive childbirth experience**. Geneva, Switzerland: World Health Organization; 2018.

2. Bohren MA, Hunter EC, Munthe-Kaas HM, Souza JP, Vogel JP, Gulmezoglu AM: **Facilitators and barriers to facility-based delivery in low- and middle-income countries: a qualitative evidence synthesis**. *Reproductive health* 2014, **11**(1):71.
