# Supplementary material for: Qualitative Evidence Synthesis (QES) for Guidelines: Paper 2 – Using qualitative evidence synthesis findings to inform evidence-to-decision frameworks and recommendations
Source: Health Res Policy Syst. 2019 Aug 8;17:75. doi: 10.1186/s12961-019-0468-4 (PMC6686513; doi:10.1186/s12961-019-0468-4)
Supplement: Supplementary file 4 — Examples of using qualitative evidence to populate the evidence-to-decision framework criterion on the acceptability of the intervention. (DOCX 21 kb) [file 12961_2019_468_MOESM4_ESM.docx]

**Additional file 4: Examples of using qualitative evidence to populate the evidence-to-decision framework criterion on the acceptability of the intervention**

| **Guideline and framework** | **Source of the findings** | **Qualitative evidence synthesis findings** | **Text developed from these finding/s for the acceptability criterion of the framework** |
| --- | --- | --- | --- |
| Expanding health worker roles to help improve access to safe abortion and post-abortion care / Medical abortion in the first trimester by pharmacists and pharmacy workers [1] | Commissioned synthesis [2, 3] | Colvin finding 22: There were some concerns among women and providers  around the potential unintended consequences of increasing access to medical abortion through self-administration with respect to women's autonomy over  their sexual and reproductive health decision-making. Specifically, there were concerns that increased access to misoprostol, especially via pharmacists, with or without prescription, could increase men's involvement in and control over abortion (either in a restrictive or a coercive fashion) and increase pressure for sex-selective abortions.  Colvin finding 24: There is distrust, however, among women and providers  in pharmacists’ ability to properly counsel and administer medical abortion. Distrust arises from their perception of pharmacists as businesspeople, as not holding adequate knowledge, and of being incapable or uninterested in  providing follow -up in the case of complications. Distrust also stems from a sense that pharmacies and pharmacists are poorly regulated and controlled thus  augmenting the potential for unequal treatment options/prices for clients and counterfeit or poor quality/”weak” drugs. | *MA1 and subtasks – Medical abortion in the first trimester by pharmacists and pharmacy workers*  ‘Three reviews suggest that acceptability among women regarding task shifting for abortion care services (including medical abortion, counselling, or abortion-related family planning services) to pharmacies was mixed: • Women sometimes preferred to go to pharmacies for information and for medical abortion because this was more convenient, private and cheaper than going to a healthcare provider (low confidence). However, women as well as health providers sometimes distrusted pharmacists’ ability to properly counsel and administer medical abortion. This distrust arose from a perception of pharmacists as businesspeople, as not holding adequate knowledge, and of being incapable or uninterested in providing follow-up in the case of complications. Distrust also stemmed from a sense that pharmacies and pharmacists were poorly regulated and controlled thus increasing the potential for unequal treatment options or prices for clients and counterfeit drugs (high confidence). • In some settings men, female friends and others purchased drugs to induce abortion from pharmacies on behalf of women (low confidence). However, men’s easy access to these drugs through pharmacies led to concern among health-care providers and others regarding the potential to coerce women. There was also some concern among healthcare providers and older women that easy access through pharmacies would increase young women’s ability to access medical abortion indiscriminately, potentially in substitution of birth control (low confidence). • One study suggests that pharmacists’ drug recommendations depended on the customer’s ability to pay, with richer people being offered more expensive drugs; and whether or not the chemist knew the customer personally (low confidence).’ p38 |
| Intrapartum care guideline - fundal pressure [4] | Commissioned synthesis (women’s findings) [5] | 1. Authentic and kind staff (High confidence). Women's willingness to engage with maternity services was enhanced when health care providers were perceived to be authentic and kind. A friendly, respectful and attentive approach was appreciated by women, especially those who were feeling worried or anxious about their pregnancy.  2. Hope for a quick labour (Low confidence). Some women want labour to be a relatively quick process and sometimes believe that the longer it lasts the more likely they are to require (unwanted) medical intervention.  3. Desire to be in control (High confidence). Women expect to exert some control over the way their labour progresses including being informed of developments, being involved in decision making and being able to manage their pain (with or without pharmacological intervention). | There is no specific evidence from the qualitative systematic reviews on either women's or providers’ experiences of receiving or applying fundal pressure. However, general findings from this document suggest that women would rather avoid this type of procedure unless their baby is at risk (high confidence in the evidence). They would also like to be cared for by competent, skilled and sensitive health professionals (high confidence in the evidence) and even though they would prefer to have a quick labour (low confidence in the evidence) they would, where possible, like to remain in control of their labour and birth (high confidence in the evidence). |

**References**

1. WHO: **Health worker roles in providing safe abortion care and post-abortion contraception**. Geneva: World Health Organization; 2015.

2. Wainwright M, Colvin CJ, Swartz A, Leon N: **Self-management of medical abortion: a qualitative evidence synthesis**. *Reprod Health Matters* 2016, **24**(47):155-167.

3. WHO: **Health worker roles in providing safe abortion care and post-abortion contraception. Web Supplement 3. Annexes 27–40: Evidence base for acceptability and feasibility**. Geneva: World Health Organization. Available at <http://apps.who.int/iris/bitstream/10665/177628/1/WHO_RHR_15.11c_eng.pdf?ua=1>; 2015.

4. WHO: **WHO recommendations: intrapartum care for a positive childbirth experience**. In*.* Geneva, Switzerland: World Health Organization; 2018.

5. Downe S, Finlayson K, Thomson G, Hall-Moran V, Feeley C, Oladapo OT: **WHO recommendations for interventions during labour and birth: Qualitative evidence synthesis of the views and experiences of service users and providers**. 2018 (Unpublished).
