# Supplementary material for: Qualitative Evidence Synthesis (QES) for Guidelines: Paper 2 – Using qualitative evidence synthesis findings to inform evidence-to-decision frameworks and recommendations
Source: Health Res Policy Syst. 2019 Aug 8;17:75. doi: 10.1186/s12961-019-0468-4 (PMC6686513; doi:10.1186/s12961-019-0468-4)
Supplement: Supplementary file 5 — How qualitative evidence has influenced the formulation of recommendations – example from the WHO antenatal care guideline. (DOCX 16 kb) [file 12961_2019_468_MOESM5_ESM.docx]

**Additional file 5: How qualitative evidence has influenced the formulation of recommendations – example from the WHO antenatal care guideline [1]**

| **Guideline question: In populations with low dietary calcium intake, should daily calcium supplementation be recommended for pregnant women to reduce the risk of pre-eclampsia?** | |
| --- | --- |
| **Acceptability** | A systematic review of qualitative research exploring women’s views and experiences of antenatal care suggests that they tend to view antenatal care as a source of knowledge and information and generally appreciate any advice (including dietary or nutritional) that may lead to a healthy baby and a positive pregnancy experience (high confidence in the evidence).  A systematic review of research exploring health professionals views of antenatal care suggests that they are keen to offer general health care advice and specific pregnancy-related information (low confidence in the evidence) but sometimes feel they do not have the appropriate training and lack the resources and time to deliver the service in the informative, supportive and caring manner that women want (high confidence in the evidence).  *Additional considerations*  Calcium carbonate tablets might be unpalatable to many women, as they can be large and have a powdery taste. This could have implications for both acceptability and compliance. The Cochrane systematic review did not distinguish between populations based on their dietary intake of calcium, so it is not known whether women with high or low dietary intake of calcium would experience the same benefits. Therefore, based on dietary calcium intake, the intervention may not be equally acceptable to all women. |
| **Feasibility** | This evidence was derived from a qualitative evidence synthesis conducted to support the guideline development.  Where there are likely to be additional costs associated with supplementation (high confidence in the evidence) or where the recommended interventions are unavailable because of resource constraints (low confidence in the evidence) women may be less likely to engage with services.  In a number of LMIC settings providers felt that a lack of resources, both in terms of the availability of the supplements and the lack of suitably trained staff to deliver nutritional information, may limit the implementation of this intervention (high confidence in the evidence).  *Additional considerations*  Providing calcium supplements to all pregnant women may be associated with logistical issues as supplements are bulky and will require adequate transport and storage facilities to maintain stock in facilities. |
| **Recommendation** | In populations with low dietary calcium intake, daily calcium supplementation (1.5-2.0 g oral elemental calcium) is recommended for pregnant women to reduce the risk of pre-eclampsia. |
| **Remarks** | - Dividing the dose of calcium may improve acceptability. The suggested scheme for calcium supplementation is 1.5–2 g daily, with the total dose divided into three doses, preferably taken at mealtimes. - As there is no clear evidence on the timing of initiation of calcium supplementation, stakeholders may wish to commence supplementation at the first ANC visit, given the possibility of compliance issues. - To reach the most vulnerable populations and ensure a timely and continuous supply of supplements, stakeholders may wish to consider task shifting the provision of calcium supplementation in community settings with poor access to health-care professionals. - The implementation and impact of this recommendation should be monitored at the health service, regional and country levels, based on clearly defined criteria and indicators associated with locally agreed targets. Successes and failures should be evaluated to inform integration of this recommendation into the ANC package. |

* Note that this table does not include all of the criteria in evidence-to-decision framework for this recommendation and remarks are selected. The complete (annotated) framework is available in the WHO guideline document [1]

**References**

1. WHO: **WHO recommendations on antenatal care for a positive pregnancy experience**. Geneva, Switzerland: World Health Organization; 2016.
